# Exploring the Distribution of 3D-Printed Simulator Designs Using Open-Source Databases to Facilitate Simulation-Based Learning Through a University and Nonprofit Collaboration: Protocol for a Scoping Review

**DOI:** 10.2196/53167

**Published:** 2024-05-27

**Authors:** Mithusha Sritharan, Samyah Siraj, Ginny Brunton, Adam Dubrowski

**Affiliations:** 1 Faculty of Health Sciences Ontario Tech University Oshawa, ON Canada

**Keywords:** simulation, three-dimensional printing, health professions education, database, simulator, simulator design, health care provider training, simulation-based education, simulation technology, open-source, databases, simulation-based learning, e-Learning, scoping review, technology, 3D printed simulators, design, 3D printing, 3D, health care training, university-based, research centers, gaps

## Abstract

**Background:**

Advancements in technology have enhanced education, training, and application in health care. However, limitations are present surrounding the accessibility and use of simulation technology (eg, simulators) for health profession education. Improving the accessibility of technology developed in university-based research centers by nonprofit organizations (NPOs; eg, hospitals) has the potential to benefit the health of populations worldwide. One example of such technology is 3D-printed simulators.

**Objective:**

This scoping review aims to identify how the use of open-source databases for the distribution of simulator designs used for 3D printing can promote credible solutions for health care training while minimizing the risks of commercialization of designs for profit.

**Methods:**

This scoping review will follow the Arksey and O’Malley methodological framework and the Joanna Briggs Institute guidance for scoping reviews. Ovid MEDLINE, CINAHL, Web of Science, and PsycINFO will be searched with an applied time frame of 2012 to 2022. Additionally, gray literature will be searched along with reference list searching. Papers that explore the use of open-source databases in academic settings and the health care sector for the distribution of simulator designs will be included. A 2-step screening process will be administered to titles and abstracts, then full texts, to establish paper eligibility. Screening and data extraction of the papers will be completed by 2 reviewers (MS and SS) for quality assurance. The scoping review will report information on the facilitation of distributing 3D-printed simulator designs through open-source databases.

**Results:**

The results of this review will identify gaps in forming partnerships with NPOs and university-based research centers to share simulator designs. The scoping review will be initiated in December 2024.

**Conclusions:**

The information collected will be relevant and useful for stakeholders such as health care providers, researchers, and NPOs for the purpose of overcoming the gaps in research regarding the use and distribution of simulation technology. The scoping review has not been conducted yet. Therefore, there are currently no findings to report on.

**International Registered Report Identifier (IRRID):**

PRR1-10.2196/53167

## Introduction

Simulation-based education (SBE) is a rapidly growing field that requires sufficient training and expertise from health care providers who play essential roles in delivering safe and adequate care to patients [1]. Integrating health care provider training in various fields of health care facilitates advancements in technology and improves the performance of health care providers to strengthen both experiential and educational practices [1]. For decades, advancements in technology, such as the introduction of medical printing and digital technology, have promoted effective education, training, and application in health care [2]. To optimize the use of technology for adequate practice and services in health care, simulation techniques have been studied to explore the potential benefits of using 3D-printed simulators to train health care providers and the subsequent benefits on patient health and safety [3]. This education-based training method tests the technical, nontechnical, and clinical skills that are critical for health care providers to apply during patient-provider interactions [3]. Enhancing the accessibility of technology developed in university-based research centers and nonprofit organizations (NPOs) by targeting the barriers that limit the evolution of modern innovations has the potential to benefit the health of many communities worldwide [2]. Most importantly, it reflects the importance of bridging the gap between theory and application in health care and educational settings as simulation has the potential to limit medical errors [3].

SBE is an educational strategy that is used to improve training and assessment for health care providers [4]. SBE offers hands-on experience by enforcing interactions with simulators to mimic real-world scenarios to develop the expertise of health care providers and improve the quality of care that patients receive [4]. SBE in health care is emerging as a crucial educational modality as it enables learners to improve their proficiency through experiential learning using 3D-printed simulators [3]. It provides the replication of a real task without impairing the time and safety of patients [4]. The purpose of 3D-printed simulators is to supplement, not replace, existing technologies to evolve the understanding of SBE in health care [5]. In addition to educational objectives, 3D-printed simulator designs can be used for implants, prosthetics, tissue or organ modeling, therapeutic testing, and also have the potential to help patients when attempting to understand their health condition by using visual models [5].

The development of effectively designed simulation technology strengthens practices associated with training and education through the use of adequate resources in university-based research centers and NPOs [6]. Currently, there is discussion surrounding the execution of SBE from small- to large-scale interventions to be used globally [7]. However, to optimize the sustainability of simulation technology, investments in training and expertise must be targeted [7]. The consistent use of SBE creates positive patient outcomes as it increases health care professionals’ confidence in real-world situations [6]. Despite the acknowledgment of experiential learning, limited information was found on targeting the barriers surrounding training and expertise to support the use of simulation techniques. The lack of research on recognizing credible solutions for health care training and experiential learning regarding patient care through simulation techniques limit the potential of transferring theory into practice [8]. For example, it was found that students of entry-level surgical technology have no experience in the operating room, making it beneficial for health systems and schools to incorporate SBE into their curriculum to allow students to have hands-on experience and an opportunity to practice before interacting with patients [7]. It is fundamental that high-quality training for health care providers is available in both low- and high-income environments to strengthen the use of SBE globally [8]. The implementation of simulation technology relies predominantly on the capacity and availability of resources to support the expansion of SBE. The cost of acquiring resources is one of the main limitations of this intervention, posing conflict with stakeholder interactions due to the expenses of SBE [6]. Although organizations are beginning to incorporate 3D printing for health care purposes, it is found that industries are not moving fast enough to supply instruments and resources to stimulate its universal use [6]. To administer this, consistent assessment of resources required for SBE, including determining what is necessary to expand the use of simulation technology, the cost of distributing resources, and identifying what resources are available, will benefit stakeholders from university-based research centers and NPOs [8].

Despite the benefits regarding the use of simulator designs in health care, the lack of implementing the optimal use and distribution of digital designs limit the evolution of 3D-printed augmented SBE globally [9]. The use of databases as an open-source network to store data and information enables the management and collaboration of simulator designs through varying repositories [10]. Databases have the potential to facilitate the process of sharing designs to improve health care training by making designs accessible to varying institutions such as university-based research centers, hospitals, and other NPOs. While 3D-printable object databases that are readily available to the public are beneficial in introducing and contributing to designs, limitations are present in terms of the distribution of the simulators due to cost, accessibility, space, time, and expertise [10]. Forming a partnership between appropriate stakeholders can support institutions that do not have the design expertise to produce their own simulator designs. Appropriate administration for the use of simulator designs by different institutions needs to be examined to optimize and strengthen the use of the simulators.

The ability to share designs of 3D-printed simulators on different platforms benefits the use of these designs primarily by regions that experience challenges in developing a stronger workforce capacity to improve the delivery of health care services. Given that there are several open-source repositories, guidelines must be met to understand the purpose and use of these databases by varying institutions to protect the intellectual property (IP) of designs [10]. Building partnerships among university-based research centers, hospitals, and NPOs may enhance the distribution of 3D-printed simulator designs using databases that are accessible across many institutions [9]. However, it requires critical attention to how these designs can be freely used by other organizations while also protecting them from being commercialized for profit. The need to modify IP laws to constant changes in technology is an important measure that must be recognized for the security and accountability of designs published on software systems [10]. The protection of repositories requires recognition surrounding the verification and identification of the initial rights holder of the designs and frameworks published on databases [10]. Currently, limited research has been conducted on the use of databases and repositories across academic institutions to share designs and their use in multi-institutional partnerships. The objective of this scoping review is to examine the current scope of literature regarding the use of databases and repositories to store and manage simulator designs among academic institutions, hospitals, and NPOs.

## Methods

### Protocol Design

#### Overview

For this scoping review, the Arksey and O’Malley [11] methodological framework will be used and will follow the five stages of conduct: (1) determining the research question, (2) identifying relevant literature, (3) selecting studies, (4) charting data, and (5) reviewing, synthesizing, and reporting the results. The scoping review will follow the Joanna Briggs Institute guidelines for conducting a scoping review [12], as well as the PRISMA-ScR (Preferred Reporting Items for Systematic Reviews and Meta-Analyses extension for Scoping Reviews) checklist (Multimedia Appendix 1 [13]) [12].

#### Stage 1: Identifying the Research Question

The purpose of this scoping review is to explore the current literature on the distribution of 3D-printed simulator designs through open-source databases and determine how to implement the sharing of designs among academic institutions, hospitals, and NPOs while preventing the commercialization of designs for profit. The research question developed based on this purpose and in consultation with the research team is: what is the nature and breadth of research examining the use of databases and repositories to store, manage, and distribute 3D-printed simulator designs among academic institutions, hospitals, and NPOs? Preliminary searches indicate that sufficient research has not been conducted on the use of databases across various institutions and between organizations. Barriers exist in protecting the external use and commercialization of designs, resulting in the limitation of using databases to share 3D-printed simulator designs. The objectives of this scoping review are to (1) identify which open-source databases can be used to build partnerships with stakeholders to address the facilitation of SBE and (2) determine how to protect IP shared using open-source databases from being commercialized.

#### Stage 2: Identifying Relevant Studies

The published literature databases that will be searched for this scoping review are Ovid MEDLINE, Web of Science, Elsevier ScienceDirect, and IEEE Xplore. Free-text codes and database-specific subject headings will be used to create the search strategy using concepts from the research question. The keywords that were extracted from the research question include “simulation,” “educational institute,” “database,” “healthcare,” “3D-printed models.” Synonyms of these keywords will also be used in the search strategy. A search strategy has been drafted on Ovid MEDLINE and is viewable in Multimedia Appendix 2. The search will involve the selection of papers based on specific criteria, such as the publication year of the paper and the paper language, to limit the searches and establish applicable information relevant to the research question.

Gray literature databases will be used to search for papers, as these may provide information on studies that are limited in published literature databases. The gray literature databases that will be searched include OpenGrey, Grey Matters, Google Scholar, and Google. The reference list of relevant papers selected for the study will also be manually searched to find additional papers relevant to the research question. Due to the current demand for SBE in health care throughout the most recent decade, a time frame of 10 years, from 2012 to 2022, will be placed on the search to enable results of papers with the latest findings on the distribution of simulation techniques using databases. In addition, the search will also be confined to papers published in the English language. The search strategy has been developed in consultation with a health science librarian as recommended by the Peer Review of Electronic Search Strategies guidelines [14].

#### Stage 3: Study Selection

A 2-step screening process will be conducted to refine the selected papers. First, titles and abstracts will be screened to identify the purpose and relevance of the publications, with papers that do not fall within the inclusion criteria being removed from the review. Second, a full-text screening will be conducted on the selected publications from the first step by applying the same inclusion criteria to select papers for the review. The PRISMA (Preferred Reporting Items for Systematic Reviews and Meta-Analyses) flow diagram will be used to summarize the screening process. The selection of papers will involve applying precise inclusion and exclusion criteria to each publication to identify applicable papers relevant to the research question and explore papers with information on the suggested limitations. To be included in the review, papers must address the usage of simulation education in health care, discuss the use of a database or digital repository, discuss the barriers that restrict the distribution of 3D-printed simulator designs, focus on postsecondary education and incorporate university-based research centers and NPOs.

Papers will be excluded if they were published before the year 2012, are not in the English language, or focus on kindergarten to grade school education.

#### Stage 4: Charting the Data

Elements of the selected papers that will be extracted are details of the author, publication year, country of publication, the purpose of the study, the study design, details of participants, sentences describing IP, and key information that addresses the research question (the database used and purpose of using the database).

#### Stage 5: Collating, Summarizing, and Reporting the Results

The results on the use of simulation technology in health care and education regarding the facilitation in distributing 3D-printed simulator designs through open-source databases will be reported in the scoping review. Information regarding the sharing of designs between NPOs while preventing the commercialization of designs for profit will also be identified. The reported information will be organized and presented in tables and graphs where necessary and will include both qualitative and quantitative data with information summarized descriptively in the text. The findings from the papers that address the distribution of 3D-printed simulator designs as well as the barriers will be synthesized and grouped into specific themes. Subthemes will be created using information from papers that is applicable to the research gap and distribution process.

### Quality Assurance

Endnote 20 (Clarivate Analytics) is a referencing management program that will be used to manage duplicate checking. EPPI-Reviewer software (EPPI Centre), used to screen, analyze, and select papers, will be used to extract and chart the data [15]. References from Endnote 20 will be imported into EPPI-Reviewer where screening and selection of papers, analysis, and reporting will be managed. To screen and chart the data, 2 reviewers will participate in the process to maximize the validity of the results. The 2 reviewers will contribute to the screening and selection of papers individually based on relevant titles and abstracts from the search results while referring to the eligibility criteria. The reviewers will discuss their findings for a subset of papers and make changes to the eligibility criteria as required with a third reviewer settling any disagreements between the primary 2 reviewers. The first reviewer will then screen the remaining references and discuss any concerns with the second reviewer. Following the screening of papers, the first reviewer will extract data from 100% of the studies, and the second reviewer will extract data from 2 random samples of 5% each from the selected studies. This will be done to ensure adequate charting quality.

### Ethical Considerations

This scoping review will identify the current use of open-source databases in academic settings and the health care sector and build upon the importance of collaborating with partners for the distribution of simulator designs to promote solutions in health care and education. The results of this scoping review will provide an understanding of the gaps in existing literature surrounding the distribution of 3D-printed simulator designs. In addition, the results of this review will demonstrate the potential barriers in establishing partnerships with NPOs and university-based research centers to share designs globally. The information collected will be relevant and useful for stakeholders such as health care providers, researchers, and NPOs for the purpose of overcoming the gaps in research regarding the use and distribution of simulation technology. The results of this scoping review will be submitted to an academic peer-reviewed journal for publication. Ethical approval is not required for this scoping review as the data are gathered from publications that are in the public domain.

## Results

The scoping review will be initiated in December 2024. The results on the implementation of simulation technology in health care and education regarding the use and distribution of 3D-printed simulator designs through open-source databases will be reported in the scoping review. Based on prior experience in a research laboratory at the Ontario Tech University, Oshawa, Ontario, Canada, partnerships were formed with organizations to share and distribute 3D-printed simulator designs using open-source databases. Due to the challenges in sharing these resources through the database, it was determined that more research needs to be conducted on this topic.

## Discussion

This scoping review will explore the current literature on the distribution of 3D-printed simulator designs through open-source databases to facilitate the sharing of designs among academic institutions, hospitals, and NPOs. The findings from this scoping review will provide an understanding of the limitations of the existing literature regarding the distribution of 3D-printed simulator designs and identify the potential barriers in establishing partnerships with NPOs and university-based research centers to share designs globally. The findings will be used to create robust partnerships with stakeholders to ensure the delivery of SBE is effective, reliable, and accessible in educational environments. This will increase the sharing of designs while building connections with organizations to address the implementation of SBE and identify the gaps in research surrounding potential solutions in health care and education.

The scoping review has not been conducted yet. Therefore, there are currently no findings regarding the distribution of 3D-printed simulator designs to report on. However, it is expected that the scoping review will help identify the barriers surrounding how to acquire resources to support the distribution of simulation technology. The scoping review will be the first to explore current literature to determine how to facilitate the distribution of simulation technology between partners such as NPOs and university-based research centers using open-source databases. Papers will be selected from 4 published literature databases and 3 gray literature databases. Preliminary searches indicate there is a lack of findings regarding the distribution of simulator designs, limiting information on the implications and comparisons to existing literature. There is also limited information on databases that are used to share 3D-printed simulator designs, which is important in order to determine how the designs can be used by potential stakeholders and expand the use of simulation technology within organizations. The inadequacy of literature that is available on databases makes it challenging to address the research question. Because the nature of the project aims to understand the breadth of research in this area, the selected papers from the scoping review will not undergo a critical appraisal process.

The findings of the scoping review will help integrate advancements in the delivery, use, and expansion of SBE. This will not only assist in building partnerships with stakeholders to investigate the planning and delivery of SBE but will also guide how to protect IP shared using open-source databases. Determining the necessary approach and resources to expand the use of simulation technology will benefit stakeholders from university-based research centers and NPOs, facilitating the distribution of simulator designs.

## Appendix

Multimedia Appendix 1 PRISMA-ScR (Preferred Reporting Items for Systematic reviews and Meta-Analyses Extension for Scoping Review) checklist.

Multimedia Appendix 2 Draft search strategy for Ovid MEDLINE.

## Abbreviations

IP: intellectual property
NPO: nonprofit organization
PRISMA: Preferred Reporting Items for Systematic Reviews and Meta-Analyses
PRISMA-ScR: Preferred Reporting Items for Systematic reviews and Meta-Analyses Extension for Scoping Review
SBE: simulation-based education
