# Supplementary material for: Exploring the Distribution of 3D-Printed Simulator Designs Using Open-Source Databases to Facilitate Simulation-Based Learning Through a University and Nonprofit Collaboration: Protocol for a Scoping Review
Source: JMIR Res Protoc. 2024 May 27;13:e53167. doi: 10.2196/53167 (PMC11165279; doi:10.2196/53167)
Supplement: Multimedia Appendix 2 [file resprot_v13i1e53167_app2.docx]

| **Line #** | **Code** |
| --- | --- |
| **1** | Simulat*.mp. |
| **2** | exp Simulation Training/ |
| **3** | "Simulation based learning".mp. |
| **4** | "Simulation-based learning".mp. |
| **5** | exp Computer Simulation/ |
| **6** | "computer simulation".mp. |
| **7** | Technolog*.mp. |
| **8** | exp Technology/ |
| **9** | exp Printing, Three-Dimensional/ |
| **10** | "Three dimensional printing".mp. |
| **11** | "3d print*".mp. |
| **12** | 1 OR 2 OR 3 OR 4 OR 5 OR 6 OR 7 OR 8 OR 9 OR 10 OR 11 |
| **13** | "Education* institut*".mp. |
| **14** | Universit*.mp. |
| **15** | Universities/ |
| **16** | Colleg*.mp. |
| **17** | Schools, Medical/ |
| **18** | Postsecondary.mp. |
| **19** | "Post secondary".mp. |
| **20** | "Academic institut*".mp. |
| **21** | “Research institut*” |
| **22** | "Academies and Institutes"/ |
| **23** | "continuing education".mp. |
| **24** | exp Education, Continuing/ |
| **25** | "Research Lab*".mp. |
| **26** | "Research and innovat* cent*".mp. |
| **27** | "research lab"/ |
| **28** | "Research cent*".mp. |
| **29** | "Innovation cent*".mp. |
| **30** | 13 OR 14 OR 15 OR 16 OR 17 OR 18 OR 19 OR 20 OR 21 OR 22 OR 23 OR 24 OR 25 OR 26 OR 27 OR 28 OR 29 |
| **31** | Hospital.mp. |
| **32** | Hospitals/ |
| **33** | "Health care".mp. |
| **34** | Healthcare.mp. |
| **35** | exp "Delivery of Health Care"/ |
| **36** | "Health system*".mp. |
| **37** | "Health service*".mp. |
| **38** | 31 OR 32 OR 33 OR 34 OR 35 OR 36 OR 37 |
| **39** | Database*.mp. |
| **40** | Database Management Systems/ |
| **41** | Repositor*.mp. |
| **42** | portal.mp. |
| **43** | 39 OR 40 OR 41 OR 42 |
| **44** | 12 AND 30 AND 38 AND 43 |
| **45** | limit 44 to (english language and yr="2012 - 2022") |
